# Amoebic forms of *Blastocystis* spp. - evidence for a pathogenic role

**DOI:** 10.1186/1756-3305-6-295

**Published:** 2013-10-11

**Authors:** Arutchelvan Rajamanikam, Suresh Kumar Govind

**Affiliations:** 1 Department of Parasitology, University of Malaya, Kuala Lumpur 50603, Malaysia

**Keywords:** *Blastocystis* spp, Protease activity, Amoebic form, Gastrointestinal

## Abstract

**Background:**

*Blastocystis* spp. are one of the most prevalent parasites isolated from patients suffering from diarrhea, flatulence, constipation and vomiting. It’s pathogenicity and pathophysiology remains controversial to date. Protease activity and amoebic forms have been reported previously in symptomatic isolates but there has been no conclusive evidence provided to correlate the protease activity and any specific life cycle stage of the parasite thus far.

**Methods:**

Symptomatic isolates with amoebic form were tested for protease activity and compared with symptomatic and asymptomatic isolates without amoebic form for 10 days culture period.

**Results:**

The present study demonstrates an elevated protease activity in cultures having a higher percentage of amoebic forms seen in symptomatic isolates. The growth curve demonstrated a significantly (p < 0.05) higher average number of parasite counts in asymptomatic compared to symptomatic isolates. Symptomatic isolates showed amoebic forms with percentages ranging from 5% to 17%. Elevated protease activity was demonstrated in isolates that had higher percentages of amoebic forms with intense bands at higher molecular weight proteases (60 – 100 kDa). As days of culture proceeded, the protease quantification also showed a steady increase.

**Conclusion:**

This study elucidates a correlation between protease activity and percentage of amoebic forms. The finding implies that these forms could play a role in exacerbation of intestinal symptoms during *Blastocystis* spp. infection.

## Background

*Blastocystis* spp*.,* a controversial anaerobic parasite, has increasingly gained a reputation for being implicated in causing flatulence, diarrhea, constipation, vomiting and skin rash [1-3]. The mechanism of pathogenicity still remains unclear. The prevalence in humans is reported to be between 0.5% [4] to 60% [5] and 40-60% in animals [2,6].

Genotypic variability has been reported to play an influential role in the pathogenicity of *Blastocystis*[7]. This is evidenced by studies showing *Blastocystis* subtype 3 being the most common subtype isolated from patients with various gastrointestinal (GI) disorders. Despite a few prevalence studies that have been carried out in different countries, which showed subtype 3 followed by subtype 1 to be the highest [7-10], its pathogenic role has not been clearly defined. The question of whether there are other factors influencing the parasite in its ability to be opportunistic in immunocompetent and immunocompromised patients [2,11] has not yet been clearly answered.

There are also reports that demonstrate the lack of correlation between the symptomatic and asymptomatic group in terms of genotype distribution [12-14] and whether this is related to factors such as age of host and genetics is still uncertain [7]. Amoebic forms, one of the life cycle stages of the parasite, have been reported to be present in higher percentages in *in vitro* cultures of symptomatic isolates compared to asymptomatic isolates [15,16]. Previously, amoebic forms were also observed in colonoscopic lavage and patients with acute diarrheal syndrome [17,18].

Proteases from *Blastocystis* spp. have been proven to be one of the important candidates that contribute to the pathogenicity of this protozoan parasite [19,20]. Proteases from *Blastocystis* spp. have been shown to be able to degrade immunoglobin A (Ig A) giving rise to the parasite’s ability to have greater virulence and colonization through the elicitation and alteration of immunological response as well as the disruption of barrier function [21-23].

To date there have been no studies to correlate the presence of amoebic forms in *in vitro* culture and protease activity of *Blastocystis* spp. The present study attempts to elucidate if there is a correlation between the percentage of amoebic forms in cultures isolated from asymptomatic and symptomatic patients to protease activity.

## Methods

### Source of *Blastocystis* spp

Parasites were obtained from random stool sample collection in a survey carried out in a particular rural area in Selangor, Malaysia. A total of 5 isolates from symptomatic (S1-S5) and asymptomatic (A1-A5) patients were continuously cultured in Jones’ medium. The symptomatic isolates originated from patients showing symptoms such as flatulence, abdominal pain, diarrhea and constipation. This information was obtained using a questionnaire. Samples with *Blastocystis* spp. were selected through direct fecal screening and cyst concentration technique to select the samples with only *Blastocystis* spp. as the sole symptom causative agent.

### Culture and purification of *Blastocystis* spp

All isolates were inoculated in Jones’ medium supplemented with 10% horse serum as described previously by Suresh *et al.*[24]. Cultures were incubated at a constant temperature of 37°C before sub-culturing into fresh medium once every 3 days. Subsequently, the cells were purified from bacterial-contaminated culture using density gradient centrifugation. The cells were pooled into one tube and washed twice with phosphate buffered saline (PBS) for 5 minutes at 500 g. Five milliliters of the cell suspension was then layered carefully onto 6 ml of Ficoll-Paque without agitation. It was then spun for 20 mins at 700 g. *Blastocystis* spp. cells with minimal bacterial contaminants found above the thick layer of yellowish white clump was then gently isolated and washed with PBS. The pellet was stored at -20°C until further use.

### Morphological study to elucidate morphological forms in culture

Isolates, maintained for a few weeks and showing consistent growth were used subsequently for the elucidation of morphological forms in culture. 10^5^ cells in vacuolar form were counted and inoculated into culture tubes containing 1 ml of Jones’ medium with 10% horse serum and maintained to determine the growth profile at 37°C. 10 of culture suspension containing the parasites was mixed with an equal volume of Trypan blue to assess cell viability. The isolates were screened under a light microscope with 40X magnification everyday for 10 consecutive days and the percentages of amoeboid cells were observed by counting the number of amoeboid forms in a random count of 100 cells [15]. Parasites from all culture tubes on day 5 were collected, purified and the respective pellets were then stored at -20°C. In a separate experiment, cell lysates of *Blastocystis* spp. isolates from all culture tubes were obtained at different periods of incubation (Day 1, 3, 5 and 7), which were then subjected to the Gelatin-SDS-PAGE and azocasein assays for the detection of protease activity using a standardized protein concentration of 0.1 mg/ml.

### Gelatin-SDS-PAGE for the detection of protease activity

Purified isolates were washed twice with 0.85% NaCl. The respective pellets were then re-suspended with 200 of lysis buffer containing 4.3 g of sucrose and 100 mM of NaCl in 1% triton-X and kept overnight at 4°C. Protein concentration was then estimated using the Bio-Rad Microassay method (Bio-Rad). Twelve percent of resolving and 5% of stacking gel were prepared. Cell lysates with standardized protein concentration were then electrophoresed as described previously [20]. The gel was then subjected to re-naturation and incubation to observe the protease activity. Subsequently, the gel was stained with 0.12% (w/v) Coomassie Brilliant Blue R-250 for 1 hour. The protease bands appear as colorless bands with a dark blue background.

### Azocasein assay for colorimetric quantification of protease activity

Parasites were purified as described previously. The cells were lysed through a series of 15 cycles of freeze and thaw. The tubes containing the respective pellets were stored at 4°C overnight. The contents of each tube were centrifuged to sediment the debris and the protein content was estimated using the Bio-Rad Microassay method (Bio-Rad). The concentration of each sample was standardized before the assay. The cell lysates were initially incubated for 10 minutes with the supplementation of DTT (2 mM) (Sigma-Aldrich) at 37°C. This activated the parasitic proteases. 5 mg/ml of Azocasein was prepared in PBS at pH 7.4. One hundred microliters of lysate was mixed with 100 of pre-heated (37°C) azocasein solution and left to incubate at 37°C for 1 hour. The reaction was stopped by the addition of 300 of ice-cold Trichloroacetic acid and the mixture was then left on ice for 30 minutes. The tubes were then centrifuged at 8000 *g* to remove the undigested azocasein and the supernant was subsequently added to 500 of NaCl (500 mM). The clear orange solution that was produced was measured at 440 nm absorbance. Trypsin and inactivated lysate were used as positive and negative controls respectively. This experiment was carried out in triplicate.

### Statistical analysis

All statistical analysis was carried out using IBM^©^ SPSS^©^ Statistics Version 21. Independent Students t-test was used to assess the difference in the number of cells and protease activity between symptomatic isolates with amoebic forms, symptomatic isolates without amoebic forms and asymptomatic isolates. Pearson Correlation Test was used to assess the correlation between protease activity and percentage of amoebic forms. A value of p < 0.05 is considered statistically significant.

## Results

**Figure 1:**
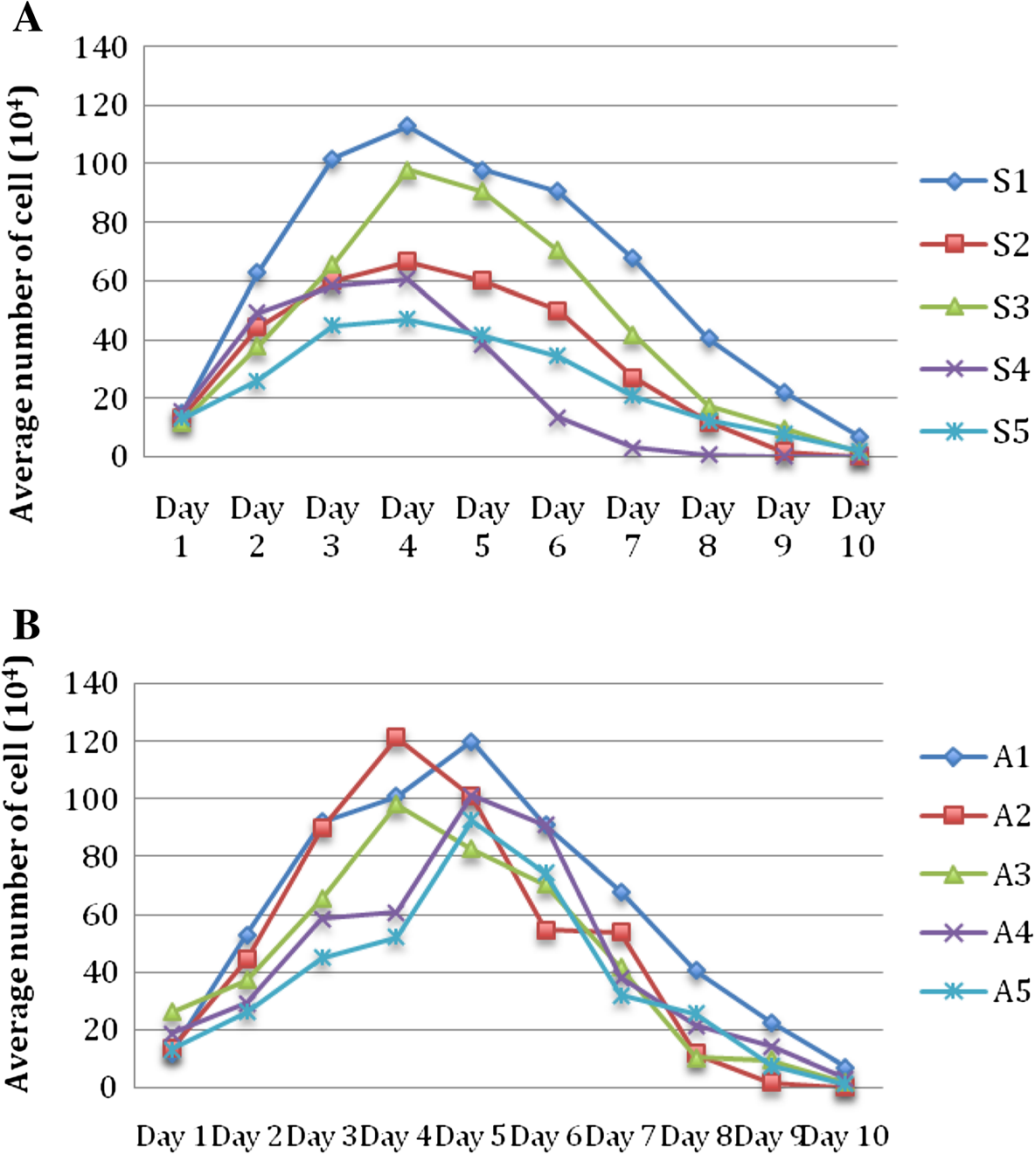
**Growth profile of *Blastocystis *spp. isolated from symptomatic (A) and asymptomatic (B) patients.** A comparison on the average number of cells is exhibited.

**Figure 2:**
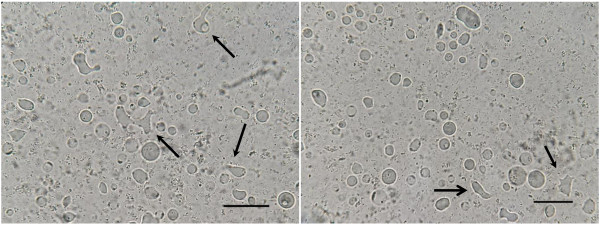
**Amoebic forms found in *in vitro *culture of symptomatic (S2) isolates on day 5.** Amoebic forms with protruding cytoplasm are indicated with arrow. Bar = 10 μm.

**Figure 3:**
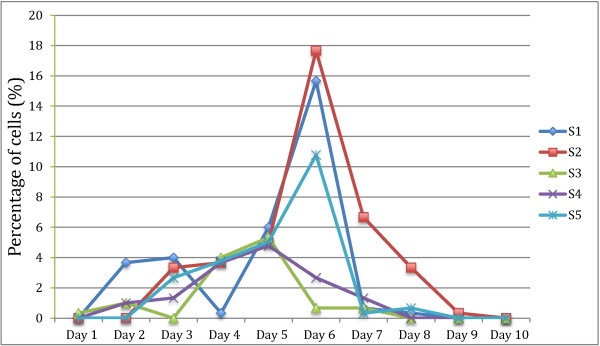
**Percentage of amoeboid forms in symptomatic isolates over a 10 days growth period.** All isolates were inoculated in Jones medium supplemented with 10% horse serum at 37C. Note: Initial culture volume was 10^5^ cells/ml.

Growth profile studies were carried out in all five symptomatic and asymptomatic isolates respectively over a 10 day period. The growth profile of both symptomatic and asymptomatic isolates for a 10 day culture period showed significantly (p = 0.008) higher peak parasite counts in asymptomatic isolates on day 5 to day 7 (Figure 1). All symptomatic isolates (S1 to S5) showed amoebic forms over the 10 day culture period with a size range between 2.9 – 8.0 μm (Figure 2). Amoebic forms were first observed on day 3 with cells showing multiple extended irregular shaped cytoplasm and a prominent nucleus. These forms persisted until day 7 (S1, S3 and S5) to day 8 (S2 and S4) with the percentage ranging from 5% to 17% (Figure 3). Higher percentages of amoebic form were observed in S2 (18%) followed by S1 and S5, which was 16% and 11% respectively. S3 and S4 showed the least number of amoebic forms. Asymptomatic isolates showed no amoebic forms in culture but had a higher parasite count with forms being mostly vacuolar followed by granular (data not shown).

Protease activity using the azocasein assay and gelatin-SDS-PAGE demonstrated higher activity in symptomatic isolates with the highest observed in S2 followed by S1, S5, S4 and S3. Asymptomatic isolates demonstrated a consistently lower activity of protease in all five isolates.

**Figure 4:**
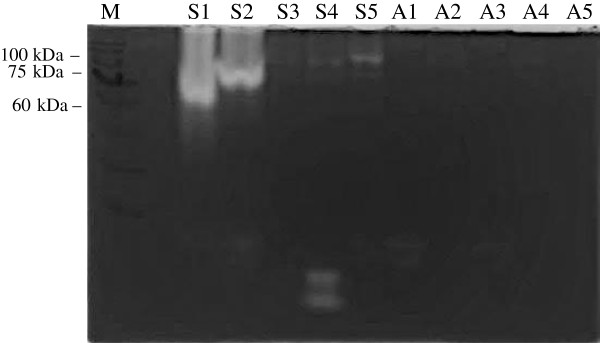
**Protease profile from the cell lysates of symptomatic isolates (S1-S5) and asymptomatic isolates (A1-A5) of *Blastocystis *spp.** The concentration of the cell lysate was standardized (0.1 mg/ml) and the lysates of each isolate was harvested on day 5 of incubation at 37C.

**Figure 5:**
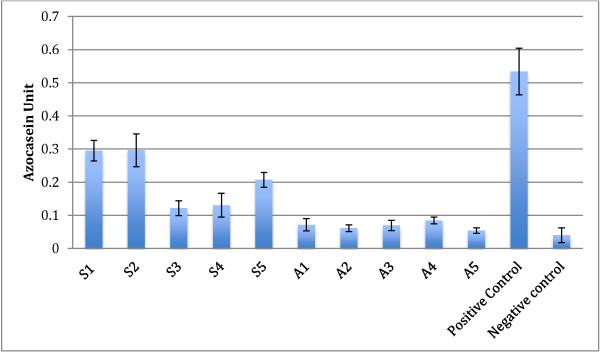
**Protease activity of symptomatic (S1-S5) and asymptomatic (A1-A5) isolates of *Blastocystis *spp. on day 5 of incubation.** The concentration of cell lysate was standardized to 0.1 mg/ml. Values are obtained from an average of three independent experiments with triplicates in each samples and standard deviations are indicated as error bars.

Elevated protease activity was demonstrated in isolates that exhibited higher percentages of amoebic forms with intense bands (Figure 4). The variation in the intensity of the band was observed only with higher molecular weight proteases (60 – 100 kDa). Protease banding patterns in current protease zymography showed a similar outcome as in the azocasein assay (Figure 5). The bands were more intense in S1, and S2 whereas the bands in S3, S4 and S5 showed less intense bands. However, only high molecular weight protease bands were observed to correlate with the percentage of amoebic forms seen in culture. Lower molecular weight protease did not show much correlation and an intense band was seen only in isolate S4 at 28–17 kDa.

**Figure 6:**
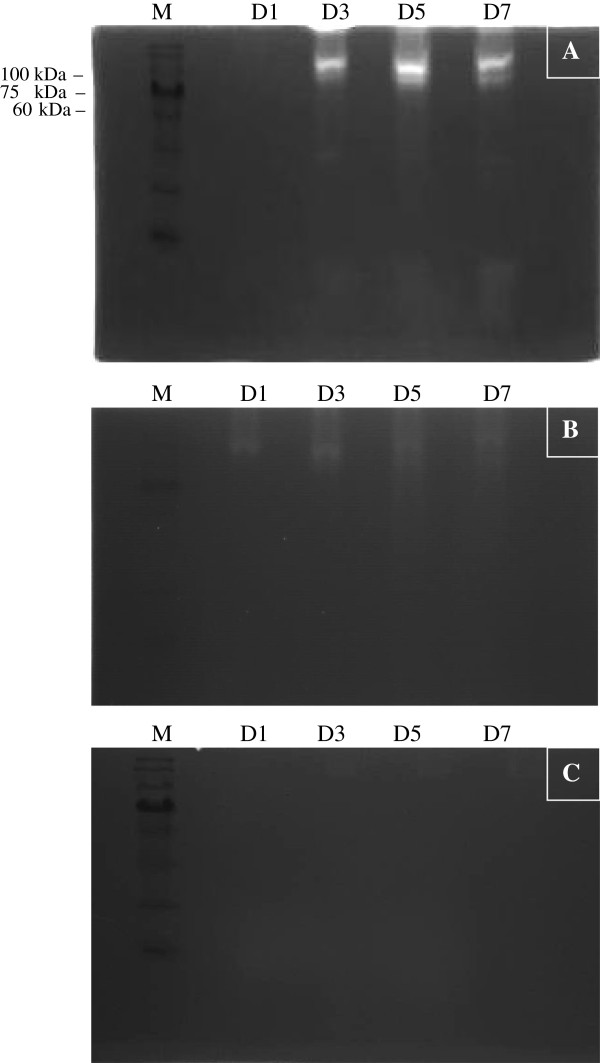
**Gelatin-SDS PAGE for protease activity of *Blastocystis *spp. isolate at 4 different culture days (D1, D3, D5 and D7). (A)** Symptomatic isolates with amoebic form. **(B)** Symptomatic isolate without the presence of amoebic form. **(C)** Asymptomatic isolates with almost no bands observed. A standard protein marker was used for all three experiments.

**Figure 7:**
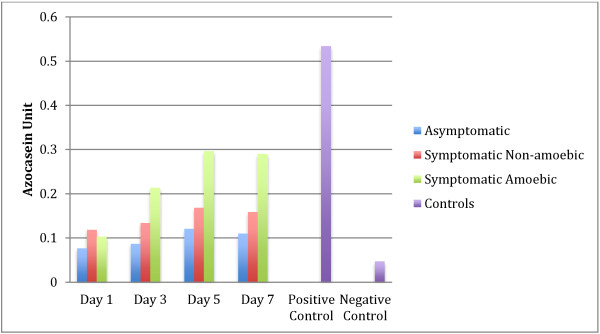
**Protease activity at 4 different points of incubation.** Trypsin was used as positive control and heat-inactivated lysate was used as negative control. Values were obtained from an average of three independent experiments with triplicates in each sample and standard deviations are indicated as error bars.

As days of culture preceded the protease quantification (Figure 6) also showed a steady increase in the band intensity until day 5 with a slight decrease observed only on day 7. Apparently, there was no obvious variation in the band intensity observed in symptomatic isolates without amoebic forms. A similar outcome was observed for asymptomatic isolates with only very faint bands spotted for all 4 different incubation days. A similar lysate concentration of symptomatic isolates with amoebic forms, symptomatic isolates without amoebic forms and asymptomatic isolates were used for the azocasein assay Figure 7. The results showed almost equal increases in protease activity on days 1, 3, 5 and 7 of the culture period. However, protease activity of symptomatic isolates with amoebic forms was significantly higher than the rest.

Symptomatic isolates with amoebic forms showed significantly higher activity of protease compared to symptomatic isolates without amoebic forms (p = 0.014) and asymptomatic isolates (p = 0.003). Furthermore, a strong correlation between the percentage of amoebic forms and average protease activity was observed (Table 1).

**Table 1:** Pearson’s correlation of average protease activity and average amoebic form count

	**p**	**Correlation coefficient(r)**
**Protease activity and amoebic cell count**	0.036	0.964

Very high correlation is observed with significance level at p < 0.05.

## Discussion and conclusions

This study is the first to report a correlation between the presence of amoebic forms and protease activity of *Blastocystis* spp. Proteases have previously been shown to be involved as one of the pathogenic factors of *Entamoeba histolytica*, *Trichomonas vaginalis* and *Acanthamoeba* sp. [25-27]. In a previous study on *Blastocystis* spp., subtype 3 of symptomatic isolates were shown to frequently posses protease activity especially at 32 kDa, which was postulated to be the virulent factor [20].

Cysteine-type activity of protease was observed in *Blastocystis* spp. isolated from patients with gastrointestinal symptoms [19] with its effect on intestinal cells and gut functions [28]. Proteases from *Blastocystis* spp. have also been shown to cleave intestinal IgA and elicit inflammatory cytokines [21,22]. However, these reports did not attempt to provide credible evidence on the life cycle stage of *Blastocystis* spp. responsible for protease activity.

The relationship of proteases to pathogenicity can be better illustrated with the example of *Entamoeba histolytica* where its membrane-associated serine proteases, with a molecular weight 60 kDa, were shown to be responsible for the degradation lysosamal proteins and tight junctions in target cells. These proteases were found in low levels in the non-pathogenic strain, *Entamoeba dispar*[29]. Proteases from *Trypanosoma cruzi*[30] and *Acanthamoeba*[31] have been shown to play a role in immune evasion during Chagas disease and be responsible for extracellular membrane degradation during granulomatous encephalitis respectively.

Amoebic forms were shown to be a life cycle stage of *Blastocystis* spp. [32] as well as being seen in cultures isolated from symptomatic patients [16,32,33]. Amoebic forms have also been reported in stool specimens of patients with diarrhea [34]. In the present study we also saw amoebic forms only in symptomatic isolates. Amoebic forms have been shown to be responsible for the engulfment of bacteria [32] which could then survive and replicate within lysosomes or phagosomes [35]. Whether bacteria contribute to the pathogenic role of the parasite still remains in question.

The present study demonstrated intense protease activity seen on day 5 in all symptomatic isolates, which correspondingly co-related to the intense bands of 60–100 kDa seen in the same day having the higher percentage of amoebic forms observed in all isolates. The presence of lower molecular weight (17 – 28 kDa) protease in isolates S2 and S4 as well as in asymptomatic isolates (A1 and A3) elicits a question on the significance of this band, which remains unclear although lower molecular weight bands in other studies have been suggested to be a virulence factor [19,20].

Moreover, our study suggests a steady increase in protease activity during the incubation corresponding to an increase in amoebic forms and a slight decrease on day 7, which relates to the drop in amoebic forms. A previous study reported that the fluctuation of cell size and protease activity appears to have a strong correlation when examined over a 96 hour incubation period [23]. This study contradicts our observation in that other than the percentage of amoebic forms, size of cell showed no influence on the protease activity. The present study involved a painstaking and tedious process to screen large samples of stools to obtain *Blastocystis* infected stool samples. We limited the study to a sample size of 5 as we had to obtain positive stool samples from symptomatic and asymptomatic patients, given that other published papers relating protease activity and *Blastocystis* spp. as well as other parasites have been carried out on a smaller sample size yet demonstrated, as in our study, significant and consistent results [19,21,27,36]. Similar studies should be carried out with larger sample sizes with isolates that have undergone genotypic characterization to statistically concrete our postulation and to completely understand the variation of protease activity in different life cycle stages of *Blastocystis* spp.

**Figure 8:**
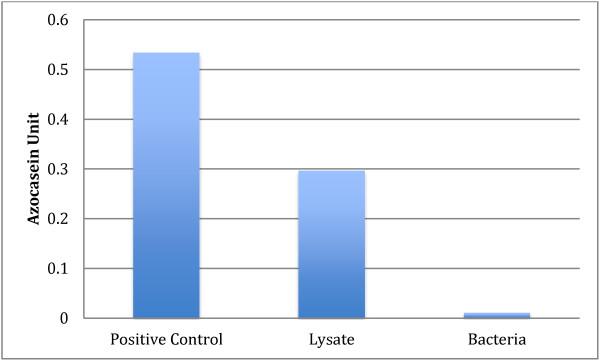
**Assessment of bacterial contamination in cell lysates of *Blastocystis *spp. used in this study.** Graph shows the protease activity of bacterial contaminant in cell lysate.

Our axenization attempts purified the parasite with minimal bacterial concentration. In the present study, lysates were extracted from purified xenic culture isolates. This was thought to mimic an environment similar to the natural intestinal microflora [15]. We have also shown that cell lysates with bacterial proteins had minimal effect since the bacterial protein demonstrated minimal protease activity (Figure 8).

The peak parasite count (parasite count between day 5 – 7) was used as one of the parameters in a previous study to differentiate the phenotypic characteristics of *Blastocystis* spp. It has been reported that asymptomatic isolates grew faster and the average total number of cells reached up to 7 times more than symptomatic isolate. The generation time was twice as high in asymptomatic isolates [37]. The present study concurred with these findings in that the asymptomatic isolates showed higher parasite peak counts than symptomatic ones (Figure 2). It is therefore evident that protease activity did not arise despite the higher parasite count consisting of mostly vacuolar and granular forms seen in asymptomatic isolates but from the larger percentage of amoebic forms seen in symptomatic isolates.

The amoebic form of *Blastocystis* spp. has been postulated previously to posses a sticky surface, which enhances the adherence to the intestinal epithelial cell lining [16]. This adherence could facilitate the release of proteases from amoebic forms to degrade extracellular membrane similar to that as seen in *Entamoeba histolytica*[38]. Higher occurrence of *Blastocystis* spp. has been seen in cancer patients undergoing chemotherapy [39]. Exacerbation of symptoms in such situations could be attributed to vacuolar stages reverting to amoebic forms, which in turn could be causing the related symptoms due to the release of proteases.

In conclusion, this is the first study to provide evidence co-relating amoebic forms of *Blastocystis* spp. and protease activity, which suggests a strong pathophysiological role that amoebic forms can play in exacerbating symptoms in patients infected with *Blastocystis* spp.

### Ethical approval

This study was approved by the Medical Ethics Committee of the University Malaya Medical Centre (UMMC) (Kuala Lumpur, Malaysia) according to the Declaration of Helsinki approved this study.

## Competing interest

Both authors declared that they have no competing interest.

## Authors’ contribution

AR and SKG were involved in the intellectual planning of the experiment; SKG designed the study; AR carried out the experimental work; AR and SKG analysed the results and wrote the paper. Both authors read and approved the final manuscript.
